# snRNA-seq of human cutaneous neurofibromas before and after selumetinib treatment implicates role of altered Schwann cell states, inter-cellular signaling, and extracellular matrix in treatment response

**DOI:** 10.1186/s40478-024-01821-z

**Published:** 2024-06-21

**Authors:** Cameron Church, Christian X. Fay, Emil Kriukov, Hui Liu, Ashley Cannon, Lauren Ashley Baldwin, David K. Crossman, Bruce Korf, Margaret R. Wallace, Andrea M. Gross, Brigitte C. Widemann, Robert A. Kesterson, Petr Baranov, Deeann Wallis

**Affiliations:** 1 Department of Genetics, The University of Alabama at Birmingham, Birmingham, AL 35294 USA; 2 Department of Ophthalmology, Harvard Medical School, Boston, MA 02114 USA; 3 The Schepens Eye Research Institute of Massachusetts Eye and Ear, Boston, MA 02114 USA; 4 Department of Molecular Genetics and Microbiology, University of Florida, Gainesville, FL USA; 5 University of Florida Health Cancer Center, Gainesville, FL USA; 6 University of Florida Genetics Institute, Gainesville, FL USA; 7 Pediatric Oncology Branch, Center for Cancer Research, National Cancer Institute, Bethesda, MD 20892 USA; 8 Department of Cancer Precision Medicine, Pennington Biomedical Research Center, Baton Rouge, LA 70808 USA

**Keywords:** Neurofibromatosis, Cutaneous neurofibroma, Selumetinib, Single nuclei sequencing, Differential gene expression, Ingenuity pathway analysis, Tumor microenvironment, CellChat, RNA velocity, Scriabin, Cell-to-cell-communication

## Abstract

Neurofibromatosis Type 1 (NF1) is caused by loss of function variants in the *NF1* gene. Most patients with NF1 develop skin lesions called cutaneous neurofibromas (cNFs). Currently the only approved therapeutic for NF1 is selumetinib, a mitogen -activated protein kinase (MEK) inhibitor. The purpose of this study was to analyze the transcriptome of cNF tumors before and on selumetinib treatment to understand both tumor composition and response. We obtained biopsy sets of tumors both pre- and on- selumetinib treatment from the same individuals and were able to collect sets from four separate individuals. We sequenced mRNA from 5844 nuclei and identified 30,442 genes in the untreated group and sequenced 5701 nuclei and identified 30,127 genes in the selumetinib treated group. We identified and quantified distinct populations of cells (Schwann cells, fibroblasts, pericytes, myeloid cells, melanocytes, keratinocytes, and two populations of endothelial cells). While we anticipated that cell proportions might change with treatment, we did not identify any one cell population that changed significantly, likely due to an inherent level of variability between tumors. We also evaluated differential gene expression based on drug treatment in each cell type. Ingenuity pathway analysis (IPA) was also used to identify pathways that differ on treatment. As anticipated, we identified a significant decrease in ERK/MAPK signaling in cells including Schwann cells but most specifically in myeloid cells. Interestingly, there is a significant decrease in opioid signaling in myeloid and endothelial cells; this downward trend is also observed in Schwann cells and fibroblasts. Cell communication was assessed by RNA velocity, Scriabin, and CellChat analyses which indicated that Schwann cells and fibroblasts have dramatically altered cell states defined by specific gene expression signatures following treatment (RNA velocity). There are dramatic changes in receptor-ligand pairs following treatment (Scriabin), and robust intercellular signaling between virtually all cell types associated with extracellular matrix (ECM) pathways (Collagen, Laminin, Fibronectin, and Nectin) is downregulated after treatment. These response specific gene signatures and interaction pathways could provide clues for understanding treatment outcomes or inform future therapies.

**Supplementary Information:**

The online version contains supplementary material available at 10.1186/s40478-024-01821-z.

## Introduction

Pathogenic variants in the *NF1* gene* (NF1)* lead to neurofibromatosis type 1 (NF1), a neurogenetic disorder affecting ~ 1:3000 individuals [1]. *NF1* encodes neurofibromin, a multidomain protein that acts as a GTPase activating protein that downregulates Ras signaling to control cellular transcription and proliferation [2]. NF1 is characterized by a variety of clinical features, including the development of benign skin lesions called cutaneous neurofibromas (cNFs) that occur due to biallelic reduction/loss of *NF1* expression in the Schwann cell lineages [3]. cNFs are abnormal growths between < 1 mm to several cm in diameter that present as soft nodules along peripheral nerves in the dermis layer of skin [4, 5]. They are observed in > 99% of adult affected individuals [6]. While cNFs do not progress to malignancy, affected individuals often consider them to be a highly burdensome feature of the disorder due to symptoms such as pain, itching, and irritation, as well as physical disfigurement that can occur due to the hundreds to thousands of cNFs that can develop [4, 7, 8].

cNFs are composed of Schwann-lineage cells, infiltrating immune cells, fibroblasts, and endothelial cells embedded in a collagenous extracellular matrix (ECM) [9–11]. Collagen makes up to 50% of the tumor’s dry weight and prior studies suggest the ECM plays a significant role in the development and maintenance of the microenvironment in cNFs [4, 10, 12–14]. Fibroblasts are responsible for ECM deposition and reorganization, and transcriptomic studies have identified collagen VI as being the most highly expressed collagen in cNFs by fibroblasts [10]. Collagen helps maintain basement membrane integrity and promotes angiogenesis and inflammation through unknown paracrine signaling mechanisms [10].

The only approved therapy for NF1 involves the downstream inactivation of Ras signaling through the MEK inhibitor selumetinib. Selumetinib has been approved to treat children with inoperable, symptomatic plexiform neurofibromas (pNFs) [15]. Therapeutic effects are primarily attributed to the inhibition of cell proliferation [16], though other mechanisms may be involved. Unfortunately, while tumors regress, they do not disappear and may regrow after treatment has stopped. Many patients experience toxic side effects, and others (about 30%) don’t respond [17]. Thus, further studies to determine why some patients don’t respond to treatment are needed. Ideally, a gene signature that correlates with either positive or negative selumetinib response could be developed, as has been done for a large and diverse panel of cell lines, could be developed [18]. While MEK activation is a prerequisite for selumetinib sensitivity, as Ras has additional effectors independent of Raf (e.g. PI3K), these effectors may mediate resistance to MEK inhibitors. Transcriptomic studies of selumetinib on human pNFs are not yet reported, though one group has evaluated the proteome of mouse pNF tumor cells treated with MEK inhibitor PD0325901 [19]. This study looked at mouse dorsal root ganglion cells cultured ex vivo and treated for 3 days with 1 ug/ml PD032590. Subsequent proteomic analysis suggests that the ECM and intercellular communication play a robust role in development of neurofibromas and that MEK inhibition results in inhibition of the ECM profile and TGF-$\upbeta$ signaling.

Currently there are no approved medical therapies for cNFs, though several are in clinical trials. We took advantage of one such clinical trial investigating cNF response to multiple monthly cycles of oral selumetinib (NCT02839720). Herein, we provide transcriptomic evaluation of matched patients pre- and on- selumetinib treated cNFs using snRNA-seq to evaluate the response to selumetinib treatment. We define cell populations, evaluate differential gene expression and pathway analysis, evaluate intrinsic and extrinsic cellular signaling through RNA velocity [20], Scriabin [21], and CellChat [22, 23] to assess how cNF tumors are impacted by selumetinib. While we find minimal changes in cellular composition (primarily involving Schwann and myeloid cells) and gene expression post- treatment, we see major differences in cell communication post-treatment. RNA velocity analysis indicates evidence for altered cell states in Schwann cells and fibroblasts post treatment, implicating them as the primary target cells for selumetinib. There are also changes in utilization of receptor ligand pairs, suggesting an important role of the ECM in cNF cellular signaling.

## Methods

### Human tissues

Human subjects and all sample collection and use were approved by the Institutional Review Board at The University of Alabama at Birmingham and conformed to NIH guidelines. Written informed consent was obtained from patients participating in clinical trial number NCT02839720. Demographics for tumor samples used for either snRNA-seq or histology are provided in Table 1. Tumors pre- and post-treatment were different because the whole tumor was removed for each sample. Information includes sex, age, tumor location (body region), change in individual tumor size post-treatment, change in size of tumors located in the same body region post treatment, change in size of all tumors measured post treatment, the number of cycles of selumetinib, dose of selumetinib, and the assay for which the tumor was utilized (snRNASeq or histology/immunofluorescence). With one exception, all on-treatment biopsy samples used in this study were obtained within 8 h of the patients receiving preceding selumetinib dose, which correlates with the expected peak selumetinib concentrations. Biopsy from patient 1 was taken off treatment for 4 weeks prior to sample collection. Due to lack of power, variability within and between patients, and unavailable measurements, no attempts were made to correlate changes in tumor size of treated samples and any changes in gene or pathway expression.

**Table 1:** Demographic Data

Patient	Sex	Age	Tumor location (Pre)	Tumor location (Post)	% Change in tumor size (Post)	% Change in size tumors in same region (Post)	% Change in size All tumors (Post)	Cycles of selumetinib	Dose (PO BID)	Assay (snRNASeq or Histology)
1	M	43	Lower Left Back	Right Middle Abdomen	− 3.55	3.94	24.8	3	75 mg	snRNASeq and Histology
2	F	74	Left Middle Back	Middle Back	NA	NA	NA	NA	50 mg	Histology
5	M	63	Abdomen	Abdomen	NA	− 30.56	− 28.78	4	75 mg	snRNASeq
			Abdomen	Abdomen	− 41.17	− 13.58	− 30.62	12	75 mg	Histology
13	F	51	Right upper thigh	Right upper abdomen	34.71	− 27.21	− 38.21	4	50 mg	snRNASeq
15	F	50	Left Ankle	Right Forearm	NA	− 47.68	− 29.17	4	50 mg	snRNASeq

### snRNA-seq of cutaneous neurofibromas

Cutaneous neurofibromas (cNFs) from 4 patients (one cNF before treatment and one on selumetinib treatment for each research participant) were snap frozen and stored at − 80 °C. Nuclei were isolated via 10 × Genomics protocol. Briefly, cNF samples were broken into finer pieces via scissors and then added to chilled lysis buffer (10 mM Tris–HCl + 10 mM NaCl + 3 mM MgCl2 + 0.1%NP40 + 0.2ug RNAse inhibitor) for 15 min with periodic mixing via wide bore tip pipets. After lysis nuclei were pelleted by centrifugation. The nuclei pellets were resuspended in wash buffer (1% BSA in PBS + 0.2ug RNAse inhibitor) and passed through a 40uM strainer to remove cellular debris and centrifuged again. Nuclei were then stained with 1–1.5ug (depending on pellet size) of TotalSeq™ anti-Nuclear Pore Complex Proteins Hashtag Antibody’s (B0451, B0452, B0453, or B0457) for 30 min at 4 °C. cNF nuclei pellets were then washed three times in wash buffer, after which the nuclei were resuspended in a final volume of 200–300 uL of wash buffer. Prior to sorting the nuclei were stained with propidium iodine and FACs sorted on a BD Aria. After sorting for a pure population of nuclei the samples proceeded to the 10 × snRNA-seq protocol.

### Data Processing

Nuclei were sequenced via Illumina’s NovaSeq6000 system, and resulting sequencing files were run through the Cell Ranger pipeline v7.1.0. Data analysis was completed using R (v 4.3.0–v 4.3.1) Seurat [24, 25] (v4.3.0–v5) and refdata-gex-GRCh38-2020-A (GRCh38 human genome reference) was used to analyze the snRNA-seq data. Nuclei were filtered based on having 200 < x < 4000 feature/genes per cell and having < 5% mitochondrial genes. All samples in each group were integrated together using Seurat for a final data set consisting of 11,545 nuclei and 30,442 identified genes. Cells were integrated based on the top 2000 differentially expressed (DE) genes in the combined dataset. Principle component analysis (PCA) was conducted to determine the number of dimensions to use for UMAP construction (based on elbow plot, 50 dimensions were used). To account for technical and sample variation, Harmony batch correction was conducted and used for downstream analysis. A UMAP was constructed, and clusters were determined using a resolution of 0.4. This yielded a total of 14 clusters. Using canonical genes from the literature and PanglaoDB database [26] for cell type identification, cell clusters were labeled manually to identify cell types (Supplemental Table 1). We observed two distinct endothelial cell populations (named endothelial cells 1 and 2), keratinocytes, melanocytes, pericytes, fibroblasts, myeloid cells, and Schwann cells. Each identified cell type was subset and DE genes for untreated and selumetinib treated were computed. Ingenuity Pathway Analysis (IPA) [27] was utilized on the differentially expressed dataset (minimum PCT 5% and minimum Log2FC |0.25| or |0.5| depending on the cell population) to determine significantly altered signaling pathways between treated and untreated cells.

### *Python* conversion and ForceAtlas2

Upon the analysis we converted the finalized.h5Seurat object to.h5ad using SeuratDisk Convert() function for further Python-dependent analysis. By connecting scanpy [28], palantir, and scFates [29] packages, we generate an advanced version of ForceAtlas2 [30] embedding. This was achieved by modifying the default functions with palantir.utils run_diffusion_maps() on the pregenerated PCA projections, and using the palantir embedding for analyzing the neighbors. Upon that, we generated a ForceAtlas2 embedding to be used in the downstream analysis.

### Cell–cell communication atlas explorer

After processing our snRNA-seq dataset in Seurat, downstream analysis in CellChat was conducted. CellChat was run using all cell types that were identified within the snRNA-seq dataset. We utilized the entire CellChat database for analysis. Due to the sequencing depth of our snRNA-seq data set (mean reads per cell 32,871–253,196) we did not use the optional function for projecting to the PPI database. In the function computeCommunProb we used default settings while changing the type parameter to “truncatedMean” and the trim parameter to 0.1. In the filterCommunication function we set the min.cells parameter to 10. After the initial CellChat objects were made for both the treated samples and control samples separately, we utilized the function getMaxWeight to directly compare the cell signaling communication pathways between the control and treated samples.

### RNA velocity

Upon performing the main Seurat processing, we used the Cell Ranger output folder to apply samtools, velocyto and scvelo packages to perform the bash- and Python-dependent analysis. samtools and velocyto were used to generate the.loom spliced/unspliced output out of the standard cell ranger.bam output. We sorted the possorted_genome_bam.bam file using samtools (-t CB -O BAM) to generate cellsorted_possorted_genome_bam.bam file. This file was used to create the.loom file with the function velocyto run10x. The reference genome used for generating the.loom file is GRRCh38, and the repeat annotation mask was obtained for the genome from the UCSC genome browser in.gtf format. The dataset in.h5seurat format was converted into.h5ad using SeuratDisk package, and the downstream analysis was performed using the scanpy and scvelo [31] packages in Jupyter Notebook. We merged.loom files with the.h5ad integrated dataset to quantify the first and second order moments (means and uncentered variances) among nearest neighbors in PCA space by utilizing the scvelo.pp.moments() function. Upon that, we estimate velocity (scvelo.tl.velocity()) and project the results with velocity embedding graph upon utilizing the scFates package ForceAtlas2 dimensionality reduction method (basis = ‘draw_graph_fa’).

### Scriabin cell–cell interactions

We performed the analysis following the standard tutorial with a few modifications. Modifications include custom relabeling the ‘sender-of-interest’ and ‘receiver-of-interest’ populations for the sender- and receiver- focused analysis. The population of interest was relabeled to be the sender of interest, to avoid the package limitation of pairwise comparison, we included all the rest of the populations by allowing them to carry the same label, and vice versa for the population of interest to be the receiver of interest.

### Statistical analysis

Two-way t-test was used for differences in cell proportions.

### Cell culture

Immortalized cutaneous neurofibroma Schwann cell lines (icNF 97.2a, icNF 97.2b, and icNF 98.4d) were obtained from Dr. Margaret Wallace. Genotypes are as follows (numbering per NM_0000267.3): icNF 97.2a: germline c.233delA; somatic c.1929delG; icNF 97.2b: germline c.233delA; somatic c.1391 + 2delTA; and icNF 98.4d: germline c.6641 + 1G > T; somatic c.6253delG. Cell lines were isolated from cNF tumors and immortalized using the normal human telomerase (hTERT) and murine Cdk4 genes as previously described [32]. Cells were cultured in Dulbecco’s modified Eagle’s medium (DMEM) + 10% fetal bovine serum, 1 × penicillin–streptomycin, and 1 × L-Glutamine using standard culture procedures. Plates were coated in 4ug/mL of laminin and incubated for 30 min before culturing.

### RT-qPCR

icNF cell lines were treated with selumetinib at 5uM and RNA was harvested after 48 h using a Qiagen RNeasy Mini kit (Qiagen, Hilden, Germany) according to the manufacturer’s directions. RT-qPCR was performed on a 384 well plate using a Luna Universal One-Step RT-qPCR kit (NEB cat# E3005) with the following QuantiTech Qiagen primers: *KCNMA1* (cat# QT00024157), *PRKCE* (cat# QT00016352), *OPCML* (cat# QT01005361), *GRIK2* (cat# QT00016597), *CADM2* (cat# QT00048888), *CREB5* (cat# QT00054488), *CAMK1D* (cat# QT00036344), and *ITGB8* (cat# QT00038507) using a Roche LightCycler480 Real Time PCR machine (Roche, Basel, Switzerland). Cycling parameters were as follows: one round of 55° C for 10 min; 45 cycles of: 95 ^o^ C for 1 min, 95 ^o^ C for 10 s, 60 ^o^C for 30 s. Relative quantification was calculated using the ddCt method with GAPDH as the internal control.

### Immunofluorescence staining

Frozen cNFs were directly embedded in Tissue-Tek® O.C.T. Compound and once frozen sectioned on a cryostat at 10 microns and collected onto slides. Frozen sections were then used for immunofluorescence staining. Briefly, sections were thawed and treated with acetone, dried and washed three times in PBS. Sections were blocked with SuperBlock™ T20 (PBS) Blocking Buffer (ThermoFisher cat# 37516) and put in primary antibody at 4 °C overnight (CD11b at 1:100 dilution (abcam Cat # ab52478) for myeloid cells and SOX10 at 1:500 dilution (abcam Cat # ab155279) for Schwann cells.) The following day slides were washed three times in PBS and then treated with secondary antibody Goat anti-Rabbit, Alexa Fluor™ 488 1:2000 and Goat anti-Rabbit, Alexa Fluor™ 594 1:2000 for 1 h at room temperature. Slides were again washed three times in PBS and mounted in anti-fade with DAPI (Invitrogen Cat#P36931) prior to sealing and imaging on Zeiss LSM 700 confocal microscope.

## Results

### snRNA-sequencing of cutaneous neurofibromas before and on- selumetinib treatment

**Fig. 1:**
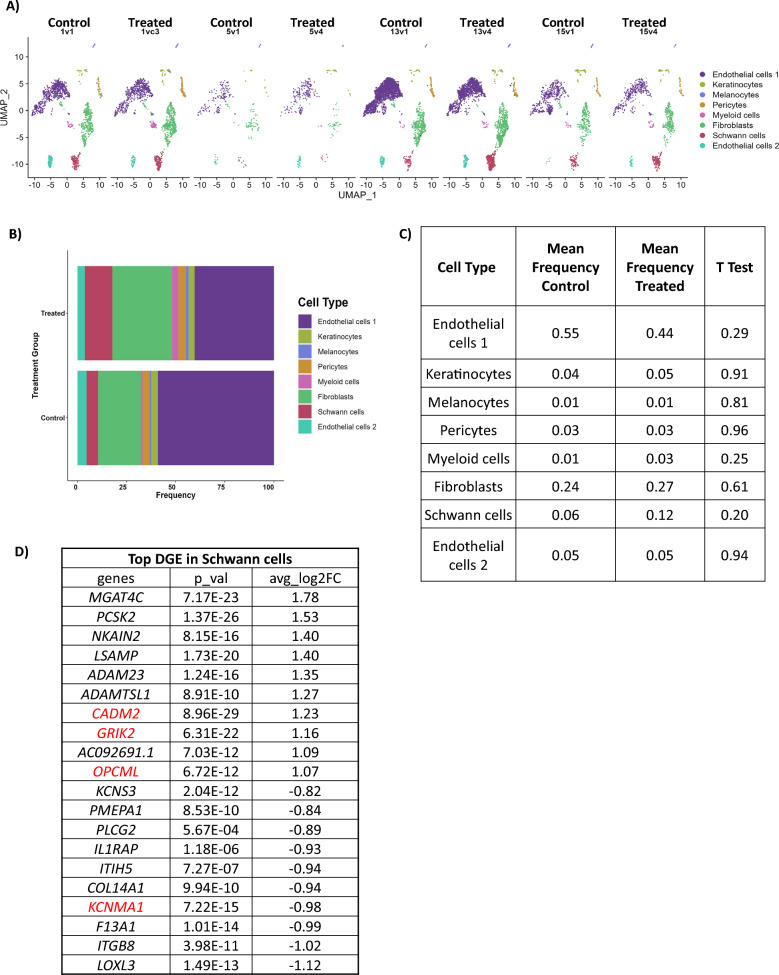
snRNA-seq of cutaneous neurofibromas pre (control or Con) and post treatment (Tx) with selumetinib. (**A**) UMAP projection of cell identities based on cell type specific gene expression for each sample. (**B**) Summative bar-graph of cell type frequency between untreated control and selumetinib treated cNFs. Cell types are color coded as per the legend. (**C**) Table lists each cell type and frequency along with value of t-test between control and treated samples. (**D**) Topmost differentially expressed genes between selumetinib treated and control Schwann cells along with significant p-values and average log2 fold changes (FC) (red lettering indicates genes selected for RT-qPCR validation). Positive log2FC indicates increased expression and negative log2FC indicates decreased expression in selumetinib treated samples compared to control

A total of 11,545 nuclei were used for analysis in Seurat. Using both mitochondrial content and number of features to filter out low quality nuclei or doublets we obtained 5,844 total nuclei and 30,442 genes from untreated tumors, and 5,701 nuclei and 30,127 genes were obtained from 4 match-paired treated tumors for a total of 8 tumors (Fig. 1A and Supplemental Fig. 1A). The nuclei were sequenced with a high read depth ranging from 32,871–253,196 mean reads per nuclei depending on the sample. Using canonical genes from PanglaoDB and literature for specific cell types, we identified cells as: Schwann cells, fibroblasts, myeloid cells, two distinct populations of endothelial cells (endothelial 1 and 2), pericytes, keratinocytes, and a population of melanocytes (Fig. 1A and Supplemental Fig. 1B). A cell frequency bar graph was created to compare the cellular composition of control and treated samples (Fig. 1B, C). Although we see variation in cell frequency based on treatment in the Endothelial 1 cells (frequency 0.548 to 0.439), Schwann cells (frequency 0.063 to 0.119), and Myeloid cell (frequency 0.01 to 0.029) populations, upon statistical analysis using a two-way t-test there were no statistically significant differences (Fig. 1C). Individual sample frequencies and fold changes between control and selumetinib treated cNFs are available in Supplemental Table 2.

### Differential gene expression (DGE)

We evaluated each cell type for DGE based on treatment. Schwann cells are presumably the most clinically relevant, and the list of the top 20 up and down regulated genes are shown in Fig. 1D. The log2 fold changes were modest; raw data for all cell types is in Supplemental Tables 3–11.

### Ingenuity Pathway analysis (*IPA*) exhibits decreases in calcium and opioid signaling post treatment

**Fig. 2:**
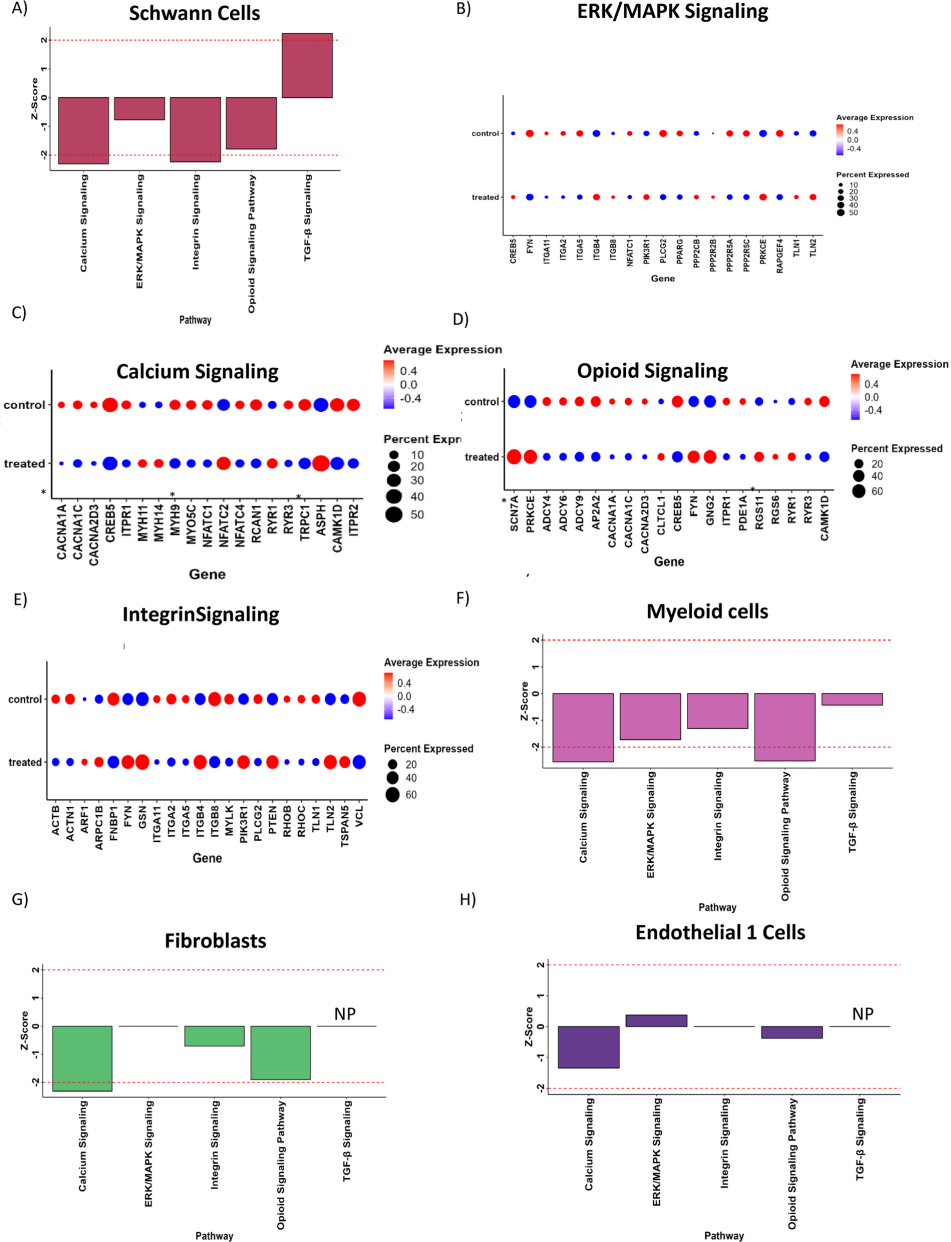
Ingenuity Pathway analysis (IPA) of selected cell populations exhibit changes in signaling. (**A**, **F**–**H**) Bar graphs showing select pathways pre- and post- selumetinib treatment in Schwann cells (**A**), myeloid cells (**F**), fibroblasts (**G**), and endothelial cells (**H**). (**B**–**E**) Dot plots for indicated signaling pathways (ERK/MAPK, Calcium, opioid, and Integrin) in Schwann cells. “NP” indicates that the pathway was not present

All cell populations were selected to assess pathway differences in the selumetinib treated tumors compared to untreated tumors. Full IPA pathway analysis for all cell types identified are available in Supplemental Table 12. While multiple pathways display significant z-scores, we call particular attention to a few pathways for each cell type. In Schwann cells, we observed a significant deactivation of “Calcium Signaling” (Z score − 2.309) and deactivation of “Opioid Signaling Pathway” (Z-score = − 1.789) (Fig. 2A). These two pathways are possibly linked. Calcium signaling is altered in patient-derived NF1 ± human keratinocytes [33] and is also increased in Nf1 ± mouse neurons [34]. Calcium signaling is involved in pain transmission and the neurofibromin–CRMP2 signaling cascade which affects calcium channel activity and regulates nociceptive neurotransmission [35]. We also see decreased “Integrin Signaling” (Z-score − 2.236). Integrins expressed in tumor cells contribute to tumor progression and integrin adhesion to the extracellular matrix (ECM) provides the traction required for malignant tumor cell invasion [36]. We observed the anticipated decrease in “ERK/MAPK signaling” as selumetinib is a MEK inhibitor, though this decrease did not reach significance in this cell type. Dot plots were obtained from differentially expressed genes for each pathway in Schwann cells (Fig. 2B–E) which allowed us to view specific genes in each pathway and the percent of cells expressing each gene. In myeloid cells we observed a significant deactivation of “ERK/MAPK signaling” and significant deactivation of “TGF-β Signaling” (Z-score = − 2.065 and − 2.333 respectively). We also saw decreased Calcium signaling (z score = − 1.941) (Fig. 2F). In fibroblasts, we again observed significant deactivation of “Calcium Signaling” (Z-score = − 2.309) (Fig. 2G). We also saw reduction in “Integrin Signaling” (Z-score = − 0.707) and Opioid Signaling (Z-score = − 1.897). While the Endothelial cells 1 population was the most frequent cell type in both control and treated tumors (Fig. 1B, C), their gene expression seems least affected by treatment, and it had reduced “Calcium Signaling” (Z-score = − 1.342) (Fig. 2H). These same pathways are depicted for all other cell types in Supplemental Fig. 2A–D. Note decreased calcium (Z-score − 2.673) and opioid signaling (Z-score − 2.197) in Endothelial 2 cells (Supplemental Fig. 2A).

To investigate specific genes involved in these pathway changes, dot plots were created from differentially expressed genes related to “Opioid Signaling Pathway” and “Calcium Signaling” in Fibroblasts (Supplemental Figs. 2E, F) and “Calcium Signaling” in myeloid cells (Supplemental Fig. 2H) and endothelial 1 cells (Supplemental Fig. 2H). These data indicated that both opioid and calcium signaling are altered in multiple cell types following selumetinib treatment and allowed us to define specific genes for later validation.

### RNA Velocity Analysis indicates selumetinib treatment alters cell states and gene signatures in Schwann cells and fibroblasts

**Fig. 3:**
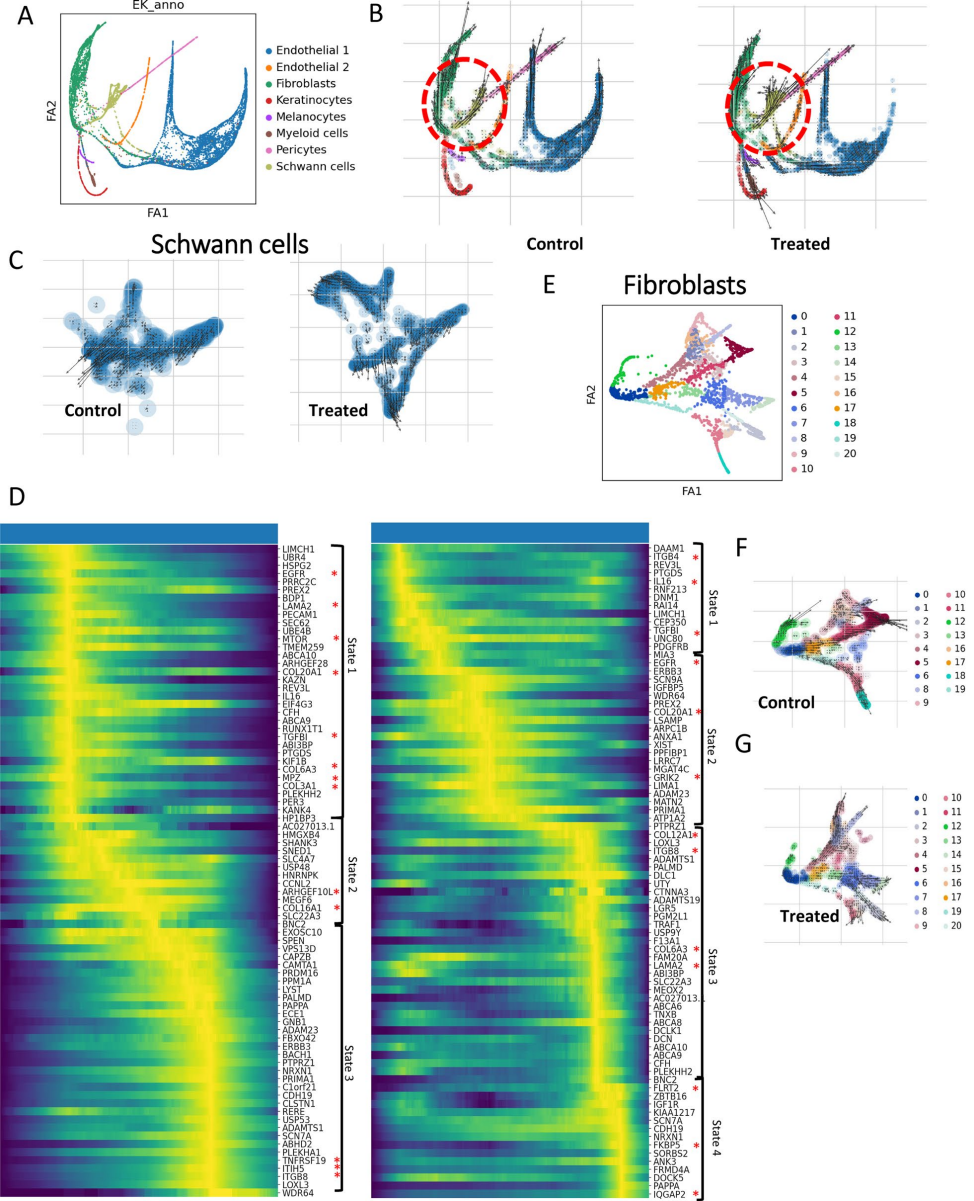
RNA Velocity Analysis indicates selumetinib treatment alters cell states and gene signatures in Schwann cells and Fibroblasts. (**A**) FA2-based dimplot of integrated non-treated and treated groups showing the major cell types present in the dataset. (**B**) RNA Velocity analysis indicates altered trajectories for Schwann cells following selumetinib treatment (red circle). Local vectors demonstrate the cell fate trajectory from early stage to late stage of response, and the vector length codes the velocity of the transition. (**C**) Schwann cells subset trajectory analysis indicates altered cell states following treatment. (**D**) Schwann cell gene expression signatures on the latent time-ordered heatmap show different conditional gene patterns between control and treated samples. Cell states are indicated by brackets. (**E**) RNA Velocity analysis of fibroblasts in untreated (**F**) vs treated (**G**) shows several distinct states and trajectories, characterized by different transcriptomic patterns, highlighting the heterogeneity of fibroblasts subpopulations

To evaluate how RNA expression and cell state changes between control and treated cells, we utilized the RNA velocity cell fate trajectory reconstruction approach. FA2-based plots were generated and utilized to view the trajectory of various cell populations (Fig. 3A). Notably the Schwann cells and the fibroblasts showed different trajectories between control and treated groups (Schwann cells are circled in red, Fig. 3B). We also observe changes for the myeloid population, although the cell number was insufficient to build the trajectories. Schwann cell subset trajectory analysis indicated altered cell states following treatment (Fig. 3C). For untreated Schwann cells, we observe two main cell states, with a short one-way transition between them for the non-treated group (Fig. 3D). Those states are characterized by the following genes: initial—*EGFR, LAMA2, MTOR, COL20A1, TGF*$\upbeta$*1, COL6A3, MPZ, COL3A1*, transitory—*ARHGEF10L* and *COL16A1*, final –*ITGB8, ITIH5,* and *TNFRSF19*. For the treated conditions we observed increased velocity of cell transformation, as well as four defined Schwann cell states. The initial state has the profile of I*TGB4, IL16,* and *TGF*$\upbeta$*1*, first transitory—*EGFR, COL20A1,* and *GRIK2*, second transitory—*COL12A1, ITGB8, COL6A3, LAMA2,* and final—*FLRT2, FKBP5,* and *IQGAP2*. For the fibroblast population, we observed heterogeneity: one (Cluster 5) and two unique branches, representing cell state trajectories in non-treated and treated groups (Cluster 2 and 14) (Fig. 3E–G). The non-treated fibroblast population (Cluster 5) is characterized by *PRRX1, COL14A1,* and *CDH11,* and the populations in the treated condition (clusters 2 and 14) carry the profile of *ZBTB16, PID1, KAZN,* and *PTPRG, RORA, DLCK1,* respectively (Supplemental Fig. 3). Our RNA velocity data indicated that both Schwann cells and fibroblast are undergoing cell state changes following selumetinib treatment.

### Scriabin analysis shows the change in cell–cell interaction upon treatment at single-cell resolution

**Fig. 4:**
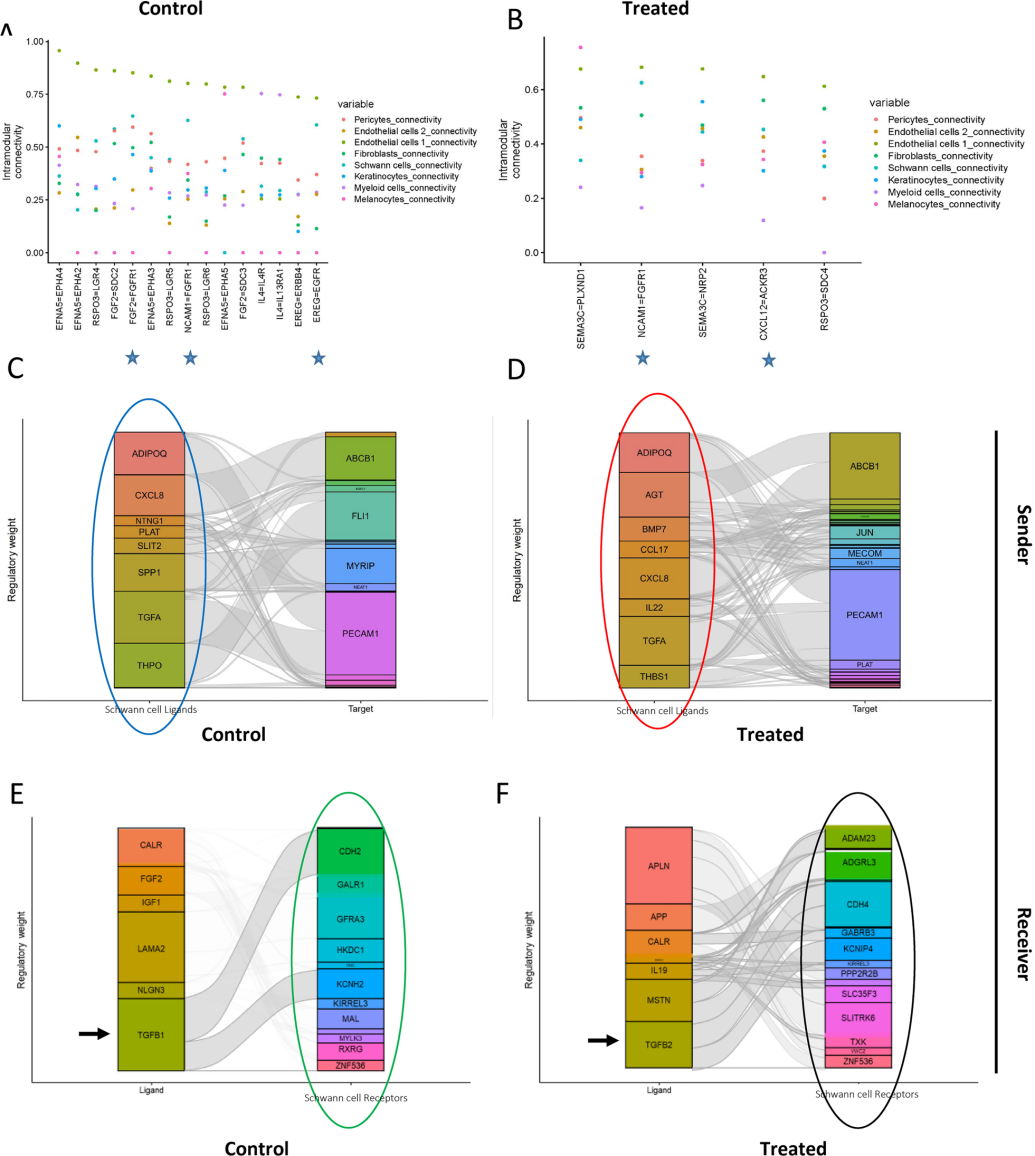
Scriabin analysis shows the change in cell–cell interaction upon treatment at single-cell resolution. Top differentially expressed ligand-receptor pairs in untreated (**A**) vs, treated (**B**). Focused analysis of Schwann cell communication at the single-cell resolution allows us to study them as senders (ligand expression, **C**, **D**) and receivers (receptor expression, **E**, **F**) in untreated (**C**, **E**) and treated (**D**, **F**). This is in comparison to all other cell types being merged

We evaluated a new single-cell-resolved interaction analysis through binning (Scriabin)—an adaptable and computationally efficient method for cell–cell communication (CCC) analysis. Scriabin dissects complex communicative pathways at single-cell resolution by combining curated ligand–receptor interaction databases, models of downstream intracellular signaling, anchor-based dataset integration and gene network analysis to recover biologically meaningful CCC edges at single-cell resolution [37]. Our cell–cell interactions analysis demonstrates that before treatment (and in comparison to other cell types) cNF Schwann cells do not express unique ligands or receptors as each is expressed in at least one other cell type (Supplemental Fig. 3). The Schwann cell population is similar to fibroblasts in terms of ligand and receptor expression (Supplemental Figs. 3 and 4A). However, selumetinib induced a major shift in the ligands-receptors profile of Schwann cells that distinguished them from other populations (Fig. 4B). The strongest connectivity for the non-treated group is shown between Schwann cells (blue-green color in Fig. 4A) and Endothelial 1 (olive color in Fig. 4A) via NCAM1-FGFR1, FGF2-FGFR1, EREG-EGFR (Fig. 4A). Upon treatment the Schwann cells lose most of the ligand-receptor pathways that interact with Endothelial 1 cells and retain only NCAM1-FGFR1 (Fig. 4B). The strongest interaction for Endothelial cells 1 (olive colored Fig. 4B) upon treatment was with Fibroblasts (green color in Fig. 4B) through CXCL12-ACKR3 axis (Fig. 4B). This interaction was not detected prior to treatment, since Schwann cells had a similar receptor-ligand profile to fibroblast at this point. The Myeloid cells (lavender colored in Fig. 4A) lose their interleukin signaling after treatment as evidenced by decrease in strength of IL4-IL4R and IL4-IL13RA1 upon treatment.

We profiled Schwann cell ligand and receptor parameters before and after treatment (Fig. 4C–F). As Scriabin analysis is mainly oriented to analyze one population that is the sender-of-interest and receiver-of-interest in the dataset, we defined “Schwann cells” and have merged all non-Schwann cell classes to be used as comparator. Before treatment Schwann cells were characterized by expression of ligands: ADIPOQ, CXCL8, TGFA, THPO (circled in blue in Fig. 4C) and receptors CDH2, GFRA3, HKDC1, KCNH2, and MAL (circled in green in Fig. 4E). Upon treatment major ligands included: ADIPOQ, AGT, BMP7, CXCL8, IL22, TGFA (circled in red Fig. 4D). Treatment induced receptors include ADAM23, ADGRL3, CDH4, KCNIP4, SLITRK6 (circled in black Fig. 4F). TGFA ligand is present both before and after treatment in Schwann cells, while the TGF$\upbeta$ program switched from TGF$\upbeta$1 to TGF$\upbeta$2 upon treatment (denoted with black arrows in Fig. 4E, F).

### CellChat details dynamic intra- and inter-cellular signaling in cNFs involving the ECM both pre- and post- selumetinib treatment

**Fig. 5:**
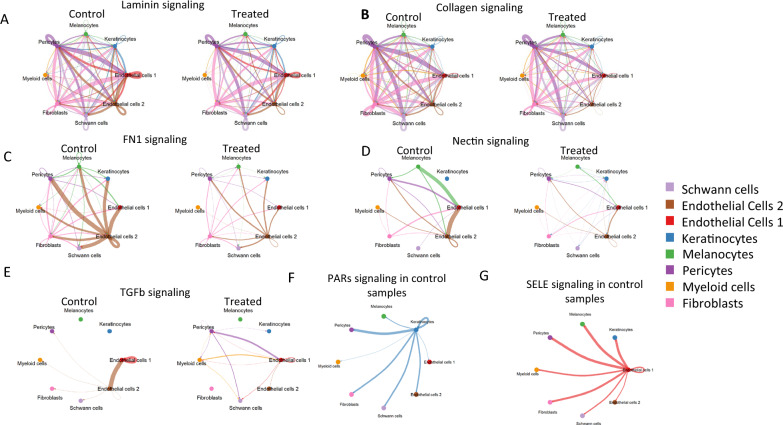
CellChat details dynamic intra- and inter-cellular signaling in cNFs involving the ECM both pre- and post- selumetinib treatment. (**A**) Laminin signaling (**B**) Collagen signaling (**C**) FN1 signaling (**D**) Nectin signaling (**E**) TGF-$\upbeta$ signaling (**F**) PARs signaling only seen in untreated controls (**G**) SELE signaling only seen in untreated controls

To get a more complete picture regarding how cells were communicating we implemented CellChat analysis. CellChat predicts major cell signaling inputs and outputs and how those cells and signals coordinate for functions using network analysis and pattern recognition approaches [22]. Summary heatmaps of incoming and outgoing signaling patterns for all cell types in both control and treated samples are provided in Supplemental Fig.4. Bar graphs at the top of each heat map indicate only modest changes between cell types overall. However, since prior literature suggests that the ECM plays a major role cNF biology, we looked specifically at these pathways. CellChat analysis indicated a significant role for cell–cell communication in ECM-related pathways in these neurofibromas, which was affected by selumetinib treatment. Consistent with neurofibromas containing significant quantities of extracellular matrix, there was robust cell adhesion signaling in our dataset detailing Laminin, Collagen, Fibronectin (FN1), and Nectin signaling to and from multiple cell types (Fig. 5A–D). While laminin signaling appeared to be robust even with selumetinib treatment, Collagen signaling was diminished slightly in treated tumors, as untreated pericytes and fibroblasts had robust connections, as indicated by purple and pink pathways, but pericytes from treated tumors did not signal as strongly to treated fibroblasts. FN1 signaling initiates from endothelial cells in untreated samples (brown pathways in Fig. 5C) but was dramatically inhibited in treated cells. Nectin signaling was also diminished in treated cells, as untreated endothelial cells send robust signals that are diminished in treated samples.

We also wanted to highlight dynamic differential signaling between control and selumetinib treated samples identified by CellChat. We saw changes in TGF-$\upbeta$ signaling (Fig. 5E). While control samples showed endothelial 1 cells receiving signaling from themselves and endothelial 2 cells, treated samples show TGF-$\upbeta$ signaling from myeloid cells and pericytes to the endothelial 1 cells, Schwann cells and keratinocytes and each other. PARS (protease activated receptors) signaling was present in untreated samples, but not detected in treated samples (Fig. 5F). PARs play a role in a multitude of physiological processes including pro-inflammatory response and pain sensation [38]. PARs may also control TGF-$\upbeta$ signaling [39, 40]. Another pathway that was observed only in the untreated samples is the SELE (E-Selectin) signaling pathway (Fig. 5G). In the control samples we observe moderate to strong signaling from the endothelial 1 cell population to all other cell types. E-selectin is a cell adhesion molecule expressed only on endothelial cells. Like other selectins, E-selectin plays an important part in inflammation. Additionally, E-selectin is inhibited by TGF-$\upbeta$ [41].

As collagens, ECM and basement membrane proteins have already been implicated to play a role in both neurofibroma development and MEK inhibitor response in mouse DRG cells[19], we evaluated our data for similarities. GO Cellular component analysis of our snRNA-seq data from Schwann cells indicates that collagen containing extracellular matrix (GO0062023), ECM (GO0031012), and extracellular region (GO0005576) all were among the top terms characterizing cNF response to selumetinib (Supplemental Fig. 5A). When we examined the expression of ECM proteins from the cellular component GO in different cell classes, we saw statistical changes (FC >|0.5| and adj P value < 0.1) in many including: ITIH5 in Schwann cells and fibroblasts, COL14A1 in all cells, Schwann cells, and fibroblasts, FN1 in endothelial cell 2, and COL4A1 in endothelial cell 2 (Supplemental Fig. 5B).

### Immunofluorescence staining of cutaneous neurofibromas and qRT-PCR analysis of cNF derived Schwann cell lines reveals no differences in cell frequencies or gene expression with selumetinib treatment

**Fig. 6:**
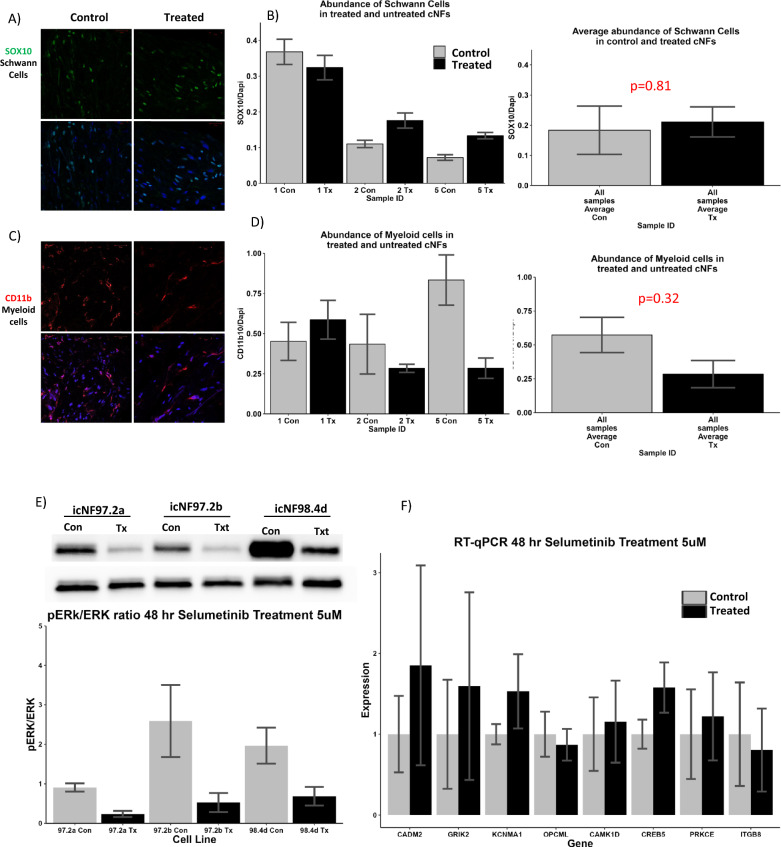
Validation of cell frequency and DGE from snRNASeq. (**A**) Immunofluorescence staining of cNFs with cell type specific marker to evaluate Schwann cell (SOX10- green) frequency between untreated and treated samples. Pictomicrographs show control samples on left and treated samples on right with Schwann cell staining both without (top) and with (bottom) DAPI nuclear stain in blue. (**B**) Histograms quantitating fluorescence; normalized to DAPI stained nuclei. Left histogram shows each tumor and right histogram shows averaged data. (**C**) Immunofluorescence staining of cNFs with cell type specific marker to evaluate Myeloid cell (CD11b-red) frequencies between untreated and treated samples. Pictomicrographs show control samples on left and treated samples on right with myeloid cell staining both without (top) and with (bottom) DAPI nuclear stain in blue. (**D**) Histograms quantitating fluorescence; normalized to DAPI stained nuclei. Left histogram shows each tumor and right histogram shows averaged data. (**E**) Western blot of lysates from human hTERT/mCdk4 immortalized Schwann cell lines derived from cutaneous neurofibromas with and without 5 uM selumetinib treatments for 48 h. Anitbodies are specific for pERK and total ERK. Histogram depicts pERK/ERK ratios of lysates pictured in Western blot based on densitometric quantification. (**F**) q RT-PCR analysis of specified genes in untreated control cells and cells treated with 5uM selumetinib for 48 h (expression on y-axis is normalized delta-delta Ct)

To investigate the differences in cell frequencies before and after selumetinib treatment, three sets of tumors were used for immunofluorescence staining to evaluate the frequency of Schwann cells and Myeloid cells in the microenvironment between control and treated samples using cell type- specific markers (SOX10 for Schwann cells (green) and CD11b for myeloid cells (red); DAPI nuclear stain (blue)) (Fig. 6A, C). Densitometry was performed using ImageJ and histograms were generated showing the quantified fluorescence for each cell type after normalization to DAPI-stained nuclei (Fig. 6B, D). As suggested by the snRNA-Seq dataset, there was no significant difference in the proportion of myeloid and Schwann cells after selumetinib treatment. Notably, there was significant variability in the number of cNF Schwann cells between the patients, with two of three patients having an increase in total Schwann cells in their neurofibromas. Similarly, there was significant variability in cNF myeloid cells based on the patient and two of three patients showed decreases in myeloid cells. Hence, variability between and within patients is large.

As Schwann cells are critical to the NF1 phenotype and formation of neurofibromas, we further assessed DGE data specifically in Schwann cells. Selected genes from the top DGE genes list (Fig. 1E, genes highlighted in red text) (*CADM2, GRIK2, KCNMA1,* and *OPCML*) and selected genes from opioid signaling (*CAMK1D, CREB5,* and *PRKCE*), calcium signaling (*CAMK1D* and *CREB5*) and Integrin signaling (*ITGB8*) (Fig. 2B) were selected for RT qPCR analysis. We utilized three different cNF immortalized Schwann cell lines from NF1 patients (icNF97.2a and icNF97.2b from one patient, and icNF98.4 from another patient). These 3 cell lines were selected as they are human Schwann cell lines that were derived specifically from cutaneous neurofibromas, similar lines are not available commercially. Cells were treated with 5 uM Selumetinib for 48 h. Despite pERK inhibition with selumetinib treatment based on Western blots (Fig. 6E), data analysis from qPCR revealed no significant difference in gene expression after selumetinib treatment of these cultured Schwann cells (Fig. 6F).

## Discussion

We identified multiple different cell types in cNF tumors including Schwann cells, fibroblasts, myeloid cells, two distinct populations of endothelial cells (endothelial 1 and 2), pericytes, keratinocytes, and melanocytes**.** One prior study has also performed single cell RNASeq of cNFs and they also identified similar cell types, though frequencies are somewhat different than our data [10]. Potential reasons for such differences include: our use of single nuclei RNA Seq instead of single cell RNASeq, our utilization of snap frozen tissues instead of fresh tissues, and the fact that our tissue digestion procedure prior to flow sorting was radically different, with ours lasting 30 min as opposed to 24 h. Further, we anticipated that selumetinib might specifically target Schwann cells for apoptosis; however, instead of decreasing in proportion following treatment, Schwann cells doubled in proportion. Further, myeloid cells tripled in proportion after treatment. None of these changes were statistically significant due to inter-tumor variability and none were replicated by immunofluorescence staining with cell type specific markers. These differences in variability may be due to the fact that different tumors from the same individual were used for both pre- or on- selumetinib treatment, and these tumors likely have differing second hit mutations to *NF1*. Additionally, as seen in Supplemental Table 2 and Fig. 6B, D, there are high degrees of inter tumor variability regarding both Schwann cells and myeloid cells from the same individual. Notably, variability within cNFs is well-described in the literature. For example, there are multiple different appearances of cNF tumors, ranging from barely visible flat nodules with subtle discoloration to large and pedunculated masses. We did not control for these differences, and it is unknown if this variability in appearance is an expression of the various stages of the evolution of a single tumor or represents different subtypes of cNF [42]. Further, there is typically dramatic variability in cNF burden between patients, and even within families harboring the same *NF1* mutation.

Our data set had high sequencing depth (32,871–253,196 mean reads per nucleus) which allowed identification of many differentially expressed genes in each cell type. However, dynamic range was lacking and few genes had large fold changes (FC >|0.5|) in expression. Taken together this suggests that only minimal changes in expression occur based on selumetinib treatment. Indeed, when we tried to validate specific genes in treated patient-derived Schwann cell lines, we were unable to document consistent changes in expression based on selumetinib treatment (Fig. 6F), despite the fact that selumetinib was clearly reducing pERK activity (Fig. 6E). These results might be due to differences in nuclear mRNA (analyzed in snRNASeq) and cytoplasmic mRNA (analyzed in q-RT-PCR). Alternatively, the immortalized nature of these cell lines could have interfered, and it is possible that the utilization of primary Schwann cells would show different results. Regardless, these findings suggest that while selumetinib treatment does alter transcription of some genes, there are other non-transcriptional mechanisms that play a significant role in modulating the effect of selumetinib treatment on cNFs. Our data suggests that cell–cell communication, specifically related to the extracellular matrix, is disrupted upon selumetinib treatment which may play a vital role in selumetinib’s mechanism of action.

We were also able to use gene set enrichment to investigate specific pathways. Many pathways were replicated across multiple cell types, suggesting that these are valid responses. We saw decreases in ERK/MAPK signaling in Schwann cells and Myeloid cells, decreases in Calcium signaling occurred in Schwann cells, myeloid cells, fibroblasts and endothelial 1 cells, decreases in Integrin signaling were seen in Schwann cells and fibroblasts, and decreases in opioid signaling in Schwann cells and fibroblasts. Each pathway is relevant to NF1 pathophysiology. Notably, we observed differences in pathways or in cell–cell communication related to pain such as calcium, opioid, and PARs signaling. Patients with plexiform neurofibromas treated with selumetinib often report decreases in pain and it is one of the most notable symptomatic improvements. Our results indicating decreased calcium and opioid signaling due to selumetinib treatment in cNFs may provide an explanation pain relief reported in patients.

RNA velocity indicated changes in cell state based on selumetinib treatment in this dataset primarily affected Schwann cells, myeloid cells, and fibroblasts. Schwann cells are desired targets, as it is their biallelic loss of *NF1* that leads to tumorigenesis. Myeloid cells (particularly mast cells) have been considered as NF1 drug targets. We have also found unique molecular signatures for fibroblast populations for control and post-treatment conditions. We had anticipated that fibroblasts might be targets, as collagen VI, a pro-tumorigenic ECM protein, is abundant in cNFs and is mainly secreted by neurofibroma fibroblasts [10], but perhaps while fibroblasts play a role in establishment of tumors and collagen deposition, they have no role in treatment response. Hence, Schwann cells and myeloid cells appear to be the primary selumetinib targets.

Scriabin cell–cell communication analysis indicated that Schwann cell ligand-receptor interactions are modulated upon treatment. We observed a major shift in ligands-receptors profile of Schwann cells that distinguishes them from other populations (Fig. 4B). Indeed, connectivity between Schwann cells and endothelial cells was drastically disrupted after treatment.

CellChat analysis indicates a very strong role for intercellular communication at the ECM level between all cells within the neurofibroma. While we did not see major differences in cell communication between control and treated tumors, we were able to detect modest down regulation of some pathways between specific cell types following treatment; this is particularly true for fibronectin, nectin, and TGF$\upbeta$ signaling. This supports our Sciabin analysis and suggests that selumetinib has a major effect on intercellular communication. Ultimately this information could be used to predict receptor and ligand interactions that might be targeted to enhance tumor response.

We had difficulty replicating gene expression changes after treatment in cell lines. Our CellChat and Scriabin data may provide an explanation. CellChat displayed a significant role of all the different cell types communicating and signaling to one another and the ECM. Scriabin showed that ligand- receptor interactions involving Schwann cells, fibroblsts, and myeloid cells were disrupted. Since we cannot replicate gene expression changes in traditional 2D immortalized Schwann cell cultures this suggests that the microenvironment and cell–cell interactions with the ECM are required for assessment of the effects of selumetinib.

These data corroborate results from proteomic analysis of mouse plexiform neurofibroma cells treated with a MEK inhibitor [19]. While both studies used different species (human vs mouse), tissues (cNFs vs pNF/Dorsal root ganglion), treatment protocols (monthly oral cycles of selumetinib vs 3 day in vitro 1 ug/ml PD0325901), and omics approaches (snRNASeq vs proteomics), they converge in indicating that ECM and intercellular communication play robust roles in development of neurofibromas. They are also consistent in showing that MEK inhibition results in inhibition of TGF$\upbeta$ signaling and disruption of intercellular communication and of the ECM. Both studies saw decreases in calcium signaling, ECM, and integrin signaling with MEKi treatment. Both studies found that collagen expression changes with MEK inhibition. This convergence of findings from radically different studies/methodologies together support a conclusion that MEK inhibition alters cell communication and ECM.

TGF-β1 signaling appears to play a role in both studies as well. Jiang et al. 2023 demonstrates that TGF-β1 signaling was identified as playing a role in ECM dynamics [19]. They showed that immune cells (macrophages and T cells) produce TGF-β1 to induce Schwann cells to produce and deposit BM proteins for ECM remodeling, and its overexpression promotes pNF progression. They also showed that following *Nf1* loss, neoplastic Schwann cells further increased BM protein deposition in response to TGF-β1. While they did not examine TGF-β1 response to MEK inhibition, our mRNA expression data showed a decrease in TGF-β1 expression in the myeloid cells (Log2FC − 0.52) (Suppl Table 4). Further, our IPA analysis showed an overall decrease in TGF-β signaling in treated myeloid cells compared to untreated samples (Fig. 2C). Finally, our Scriabin and CellChat data also identified differential TGF-β1 signaling between cells after treatment (Fig. 5E). Thus, alterations in TGF-β1 signaling appear to play roles in both neurofibroma development and maintenance after selumetinib treatment.

There are several limitations of this study. Unfortunately, we are unable to associate tumor size with treatment effects (molecular signatures, cell frequencies, pathway difference, etc.) as this data is not always available for the specific tumors assessed in this manuscript. This study included only four matched sets of tumors from four individuals for pre- and on selumetinib treatment. A larger sample size might reduce variability. While matching tumor sets may help control some variables such as background genomics and the same germline variant, matched tumor sets still have a different second hit or allelic inactivation (loss of heterozygosity) of *NF1*. We also did not define or control for either cNF tumor types or NF1 variant types (i.e. missense, nonsense, deletion, etc.) for either germline or somatic variants. Finally, we did not control for age of onset, growth rate at time of study initiation, tumor volume pre- or post-treatment, or tumor response to selumetinib.

## Conclusion

We find that while cNFs have well-described and extensive ECM, they also have an active microenvironment with all cell types signaling to each other. This signaling is particularly robust for extracellular matrix signaling pathways. Treatment with selumetinib alters cell state and signaling primarily in Schwann cells, fibroblasts, and myeloid cells. Each cell type displays a unique gene signature and ligand- receptor interactions upon response to selumetinib that upon future study and validation may be able to predict or enhance tumor response.

### Supplementary Information

Additional file 1.

Additional file 2.

Additional file 3.

Additional file 4.

Additional file 5.

Additional file 6.

Additional file 7.

Additional file 8.

Additional file 9.

Additional file 10.

Additional file 11.

Additional file 12.

Additional file 13.

## Data Availability

All data reported in this paper will be shared by the lead contact upon request. All original code has been deposited at GitHub and is publicly available as of the date of publication. DOIs are listed in the key resources table. To provide the possible updates, troubleshooting, and code optimizations, we have GitHub repository available at https://github.com/Deeann-Wallis-Lab/snRNA-seq-of-cNF. The scRNA-seq datasets are deposited in the Synapse and Genome Expression Omnibus under the accession number GEO: GSE263046. The code and processed annotated datasets generated in this study for reproducing the bioinformatic analysis can be found on the following GitHub repository: https://github.com/Deeann-Wallis-Lab/snRNA-seq-of-cNF. DOIs are listed in the key resources table. Any additional information required to reanalyze the data reported in this paper is available from the lead contact upon request. The datasets are also publicly available in the format of raw and processed data, and the code, at https://www.bnv-lab.org/data.
