# Supplementary material for: snRNA-seq of human cutaneous neurofibromas before and after selumetinib treatment implicates role of altered Schwann cell states, inter-cellular signaling, and extracellular matrix in treatment response
Source: Acta Neuropathol Commun. 2024 Jun 21;12:102. doi: 10.1186/s40478-024-01821-z (PMC11191180; doi:10.1186/s40478-024-01821-z)
Supplement: Supplementary file 13 — Additional file 13. [file 40478_2024_1821_MOESM13_ESM.pptx]

## Slide 1
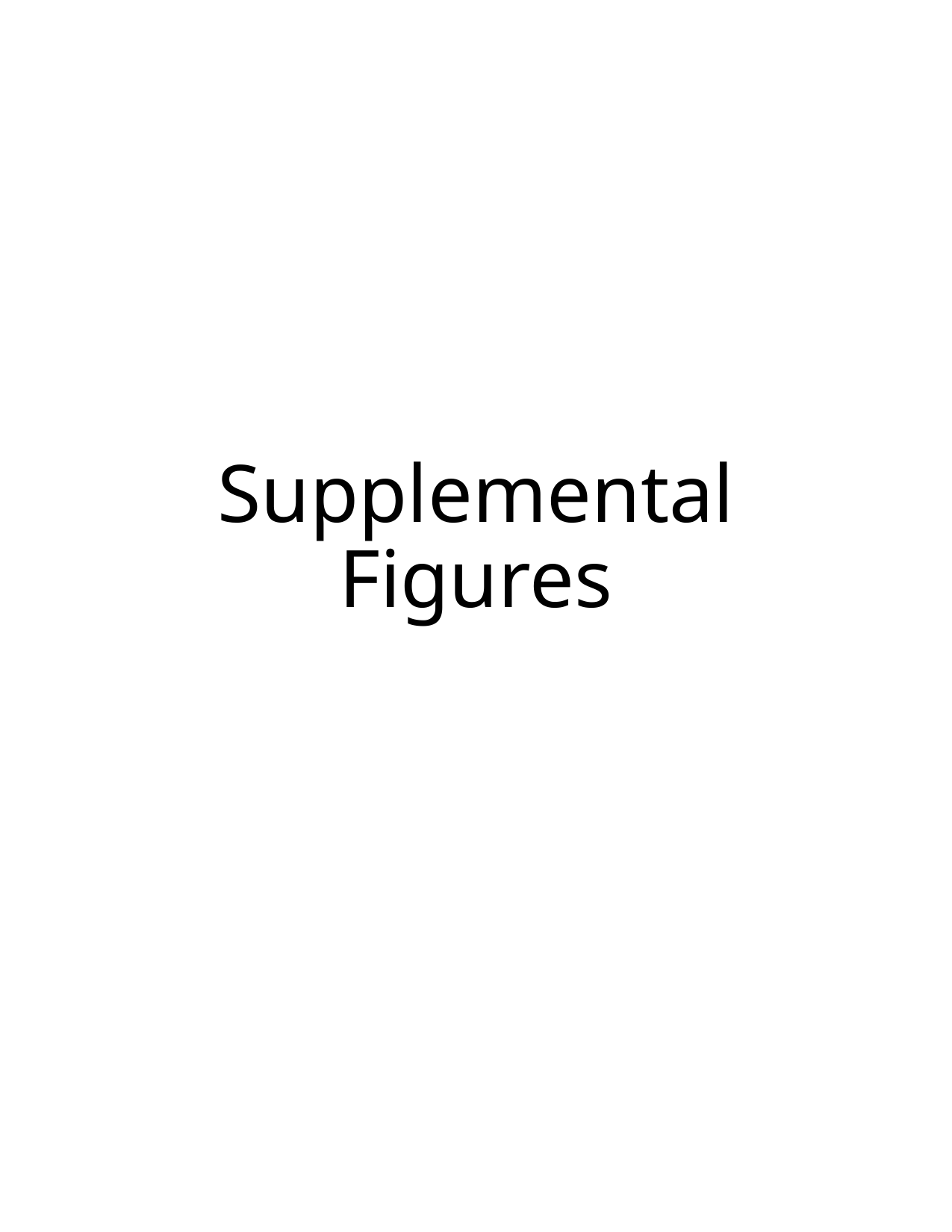

# Supplemental Figures

## Slide 2
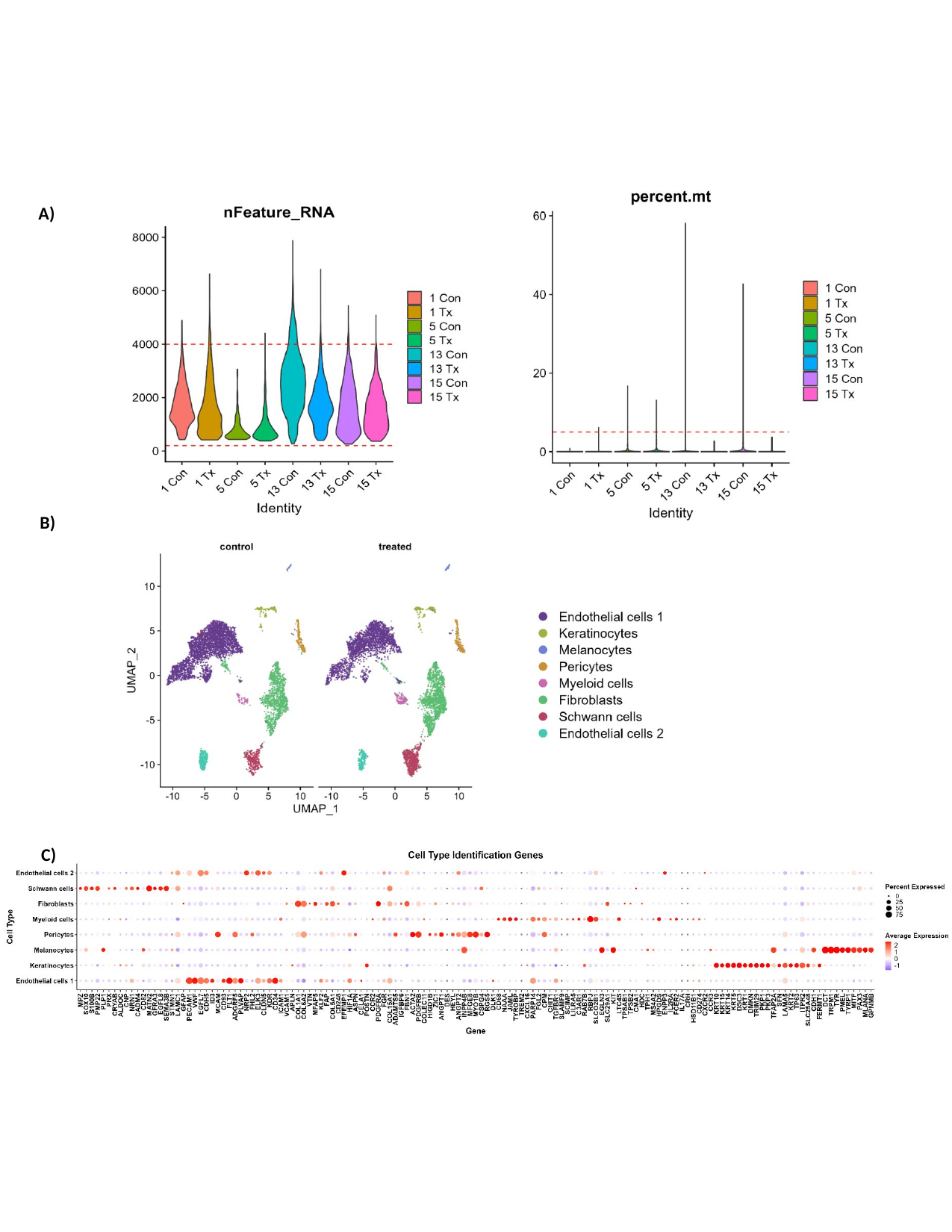

A)
B)
C)

## Slide 3
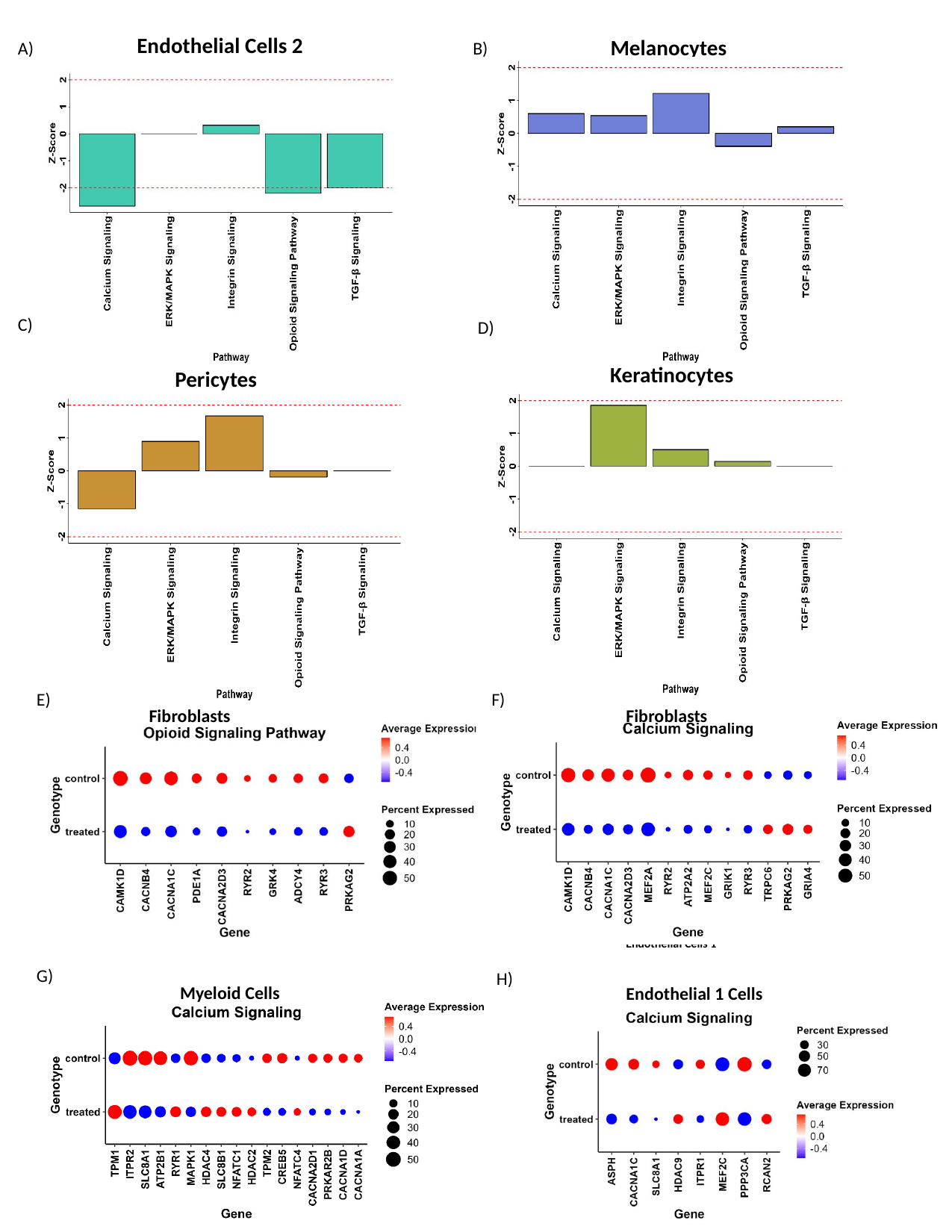

Endothelial Cells 2
Melanocytes
B)
A)
C)
D)
Keratinocytes
Pericytes
F)
E)
Fibroblasts
Fibroblasts
Myeloid Cells
Endothelial Cells 1
G)
H)
Myeloid Cells
Endothelial 1 Cells

## Slide 4
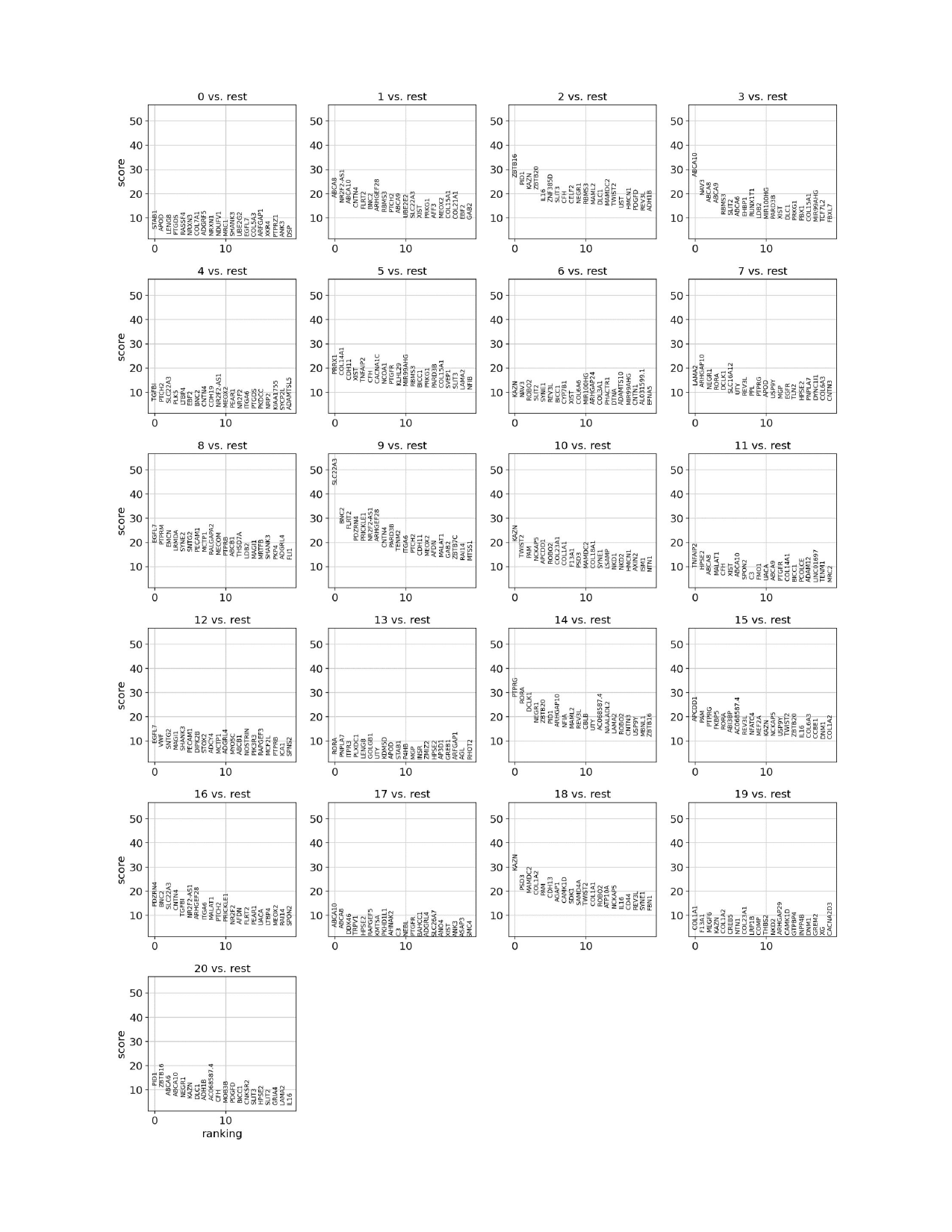

## Slide 5
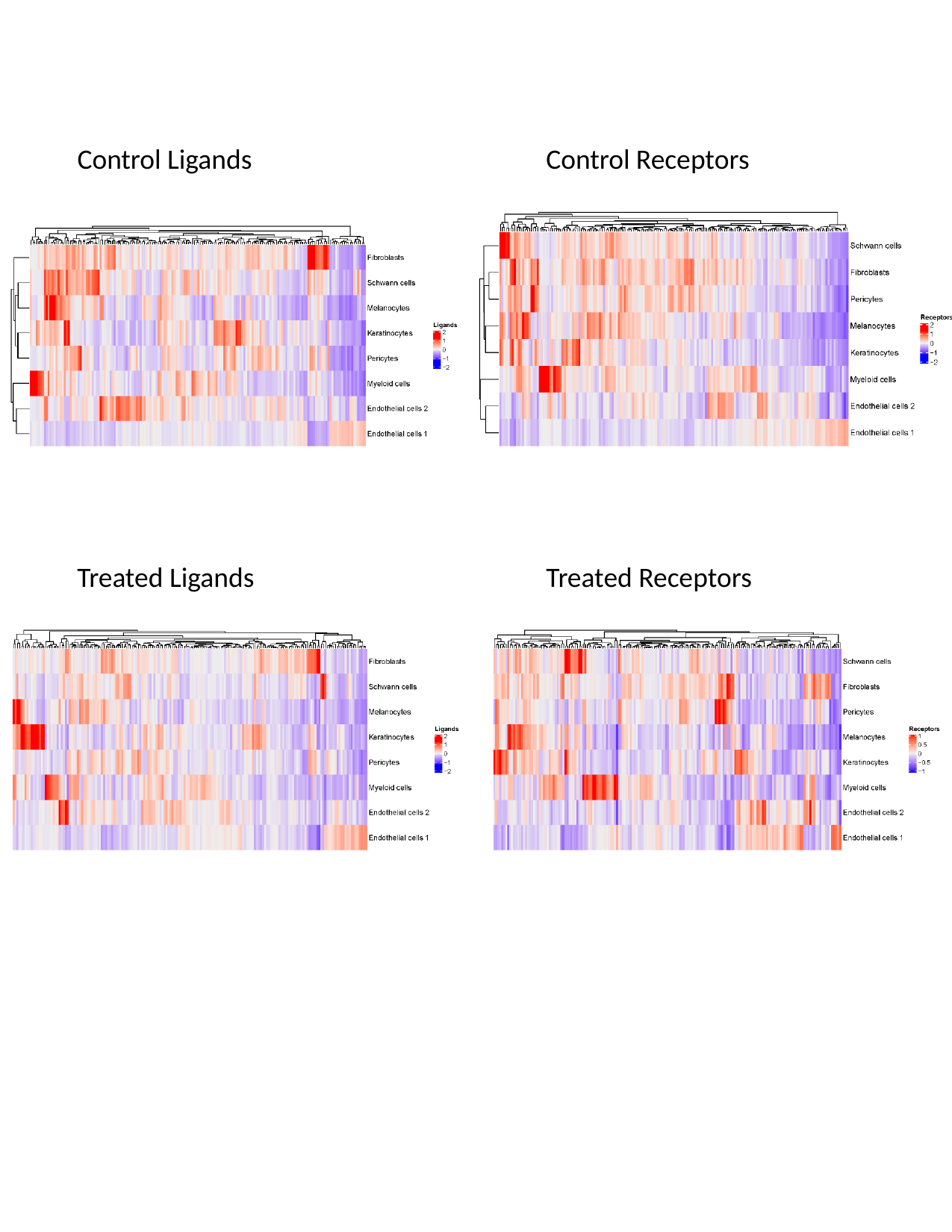

Control Ligands
Control Receptors
Treated Ligands
Treated Receptors

## Slide 6
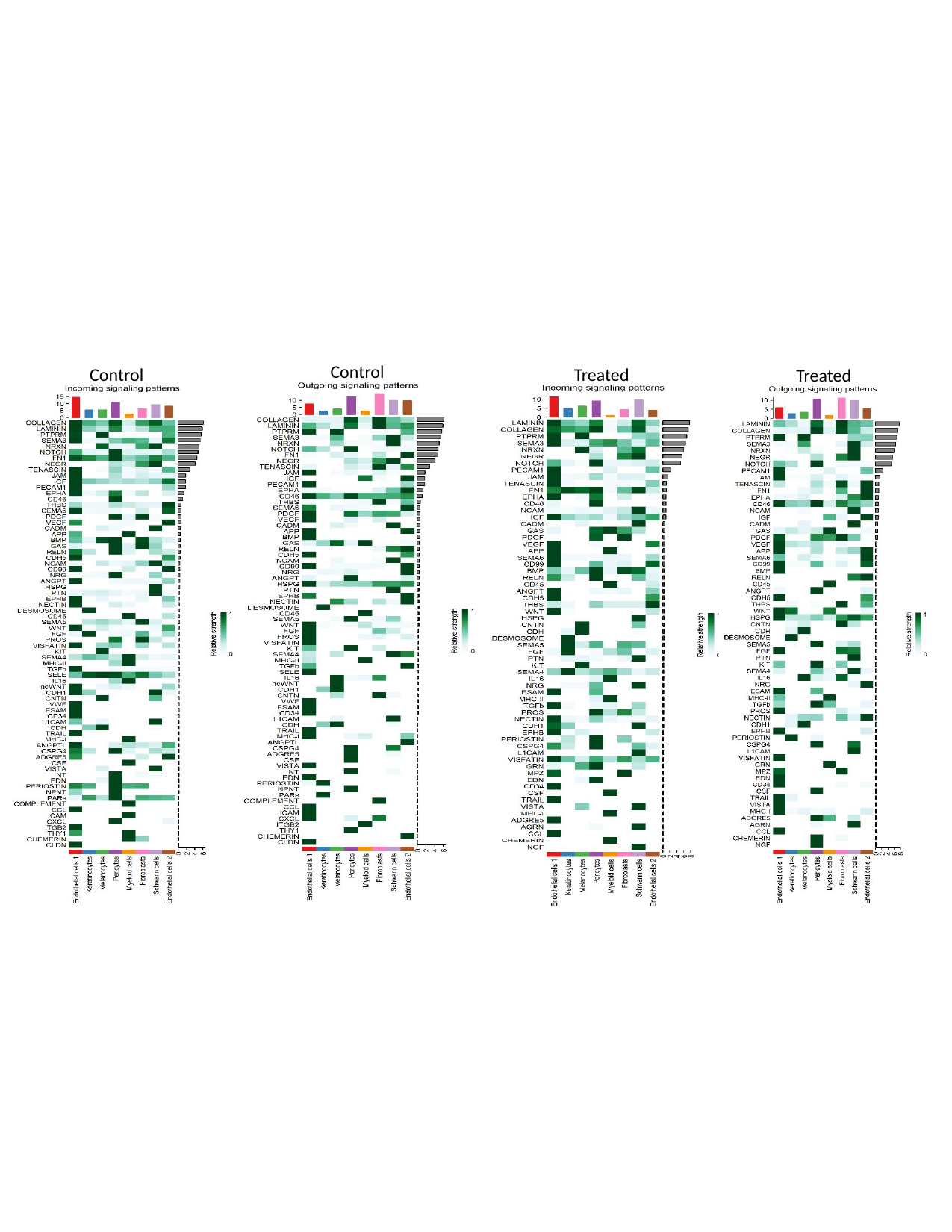

Control
Control
Treated
Treated

## Slide 7
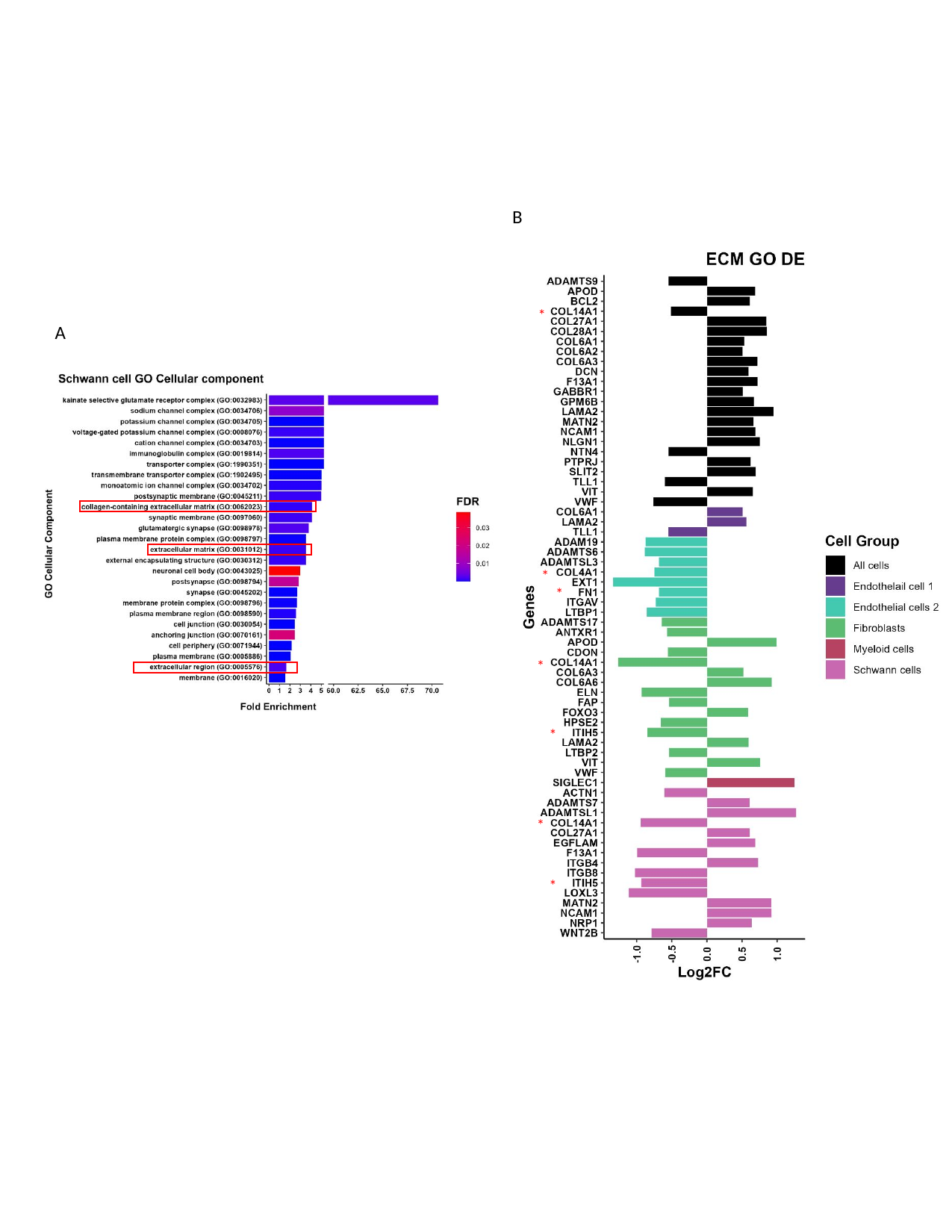

B
*
A
*
*
*
*
*
*
